# WEARABLES IN DEMENTIA RESEARCH: A SYSTEMATIC REVIEW OF BARRIERS AND FACILITATORS TO ADHERENCE

**DOI:** 10.1093/geroni/igad104.2211

**Published:** 2023-12-21

**Authors:** Colleen Peterson, Carol Flannagan

**Affiliations:** University of Michigan, Ann Arbor, Michigan, United States; University of Michigan, Ann Arbor, Michigan, United States

## Abstract

With the rapid expansion of wearable technologies, there is increased interest in their utility for passive data collection applications in aging research. Wearables can be specifically beneficial to research featuring people with dementia and their families who have burdens that can make both study participation and reliable data collection more difficult, especially as dementia progresses. For example, population-specific issues may include remembering to wear or charge a device, fluctuating acceptance/consent, and reliance on strained caregivers with other care priorities. Better understanding of how wearables can address these and other issues to enhance participant buy-in and sustained wearable use is necessary to enhance dementia research quality. Therefore, we undertook a systematic evaluation of contemporary wearables research to describe this population’s study- and non-study based wearables experiences, desired qualities, and protocol needs. We searched three databases for papers published since 2016 featuring the use or discussion of wearable devices for persons with dementia and related stakeholders (e.g., caregivers, residential care staff). Following removal of duplicates and inclusion of additional references identified through a reference/literature scan, we screened 1436 abstracts and retained 70 studies after a full-text review. We present synthesized preferences, barriers, and facilitators to wearables use and adherence in dementia research from study process analyses, interviews, or other survey measures, highlighting commonalities amongst different devices and study types. These findings inform researcher guidelines for wearable device selection and protocol design to enhance buy-in and adherence amongst persons with dementia and their caregivers.

